# Editorial: The role of empathy in behavioral change toward ethical consumption and environmental sustainability

**DOI:** 10.3389/fpsyg.2026.1857923

**Published:** 2026-05-13

**Authors:** Augusta Gaspar, Constança Carvalho, Florence Gaunet, Francisco Esteves

**Affiliations:** 1 Universidade Católica Portuguesa, Faculdade de Ciencias Humanas, Lisbon, Portugal; 2 ISPA - Instituto Universitário, Lisbon, Portugal; 3 Centre National de la Recherche Scientifique, Paris, France

**Keywords:** cruelty-free consumption, emotions, empathy, environmental sustainability, ethical behavior, nature, nature-awe, pro-environmental behavior change

Understanding the role of specific variables that cause both desirable and undesirable behaviors and using that knowledge to modify behavior have been core objectives of Psychology since its early days as a science. Nevertheless, this discipline is still struggling to achieve more than modest success in the behavior change domain. Launching “*The Role of Empathy in Behavioral Change Toward Ethical Consumption and Environmental Sustainability*” was a challenge that called for contributions that brought to light the potential of affective and cognitive experiences of empathy in driving behavioral change related to two pressing issues, ethical consumption and environmental sustainability.

Expectations were high for numerous empathy-related contributions for two reasons: (1) empathy—particularly empathic concern—is a key predictor of prosocial behavior, including pro-environmental behavior (PEB) (Gaspar, 2016; Raymond et al., 2025), and it is also a central mechanism that explains altruism beyond kinship and reciprocity (Batson et al., 2015; de Waal, 2008; Sun et al., 2025); (2) ethical behavior aligns with empathy, as demonstrated by the connection between the belief in animal minds and reduced meat consumption (Loughnan et al., 2010); however, this behavior is often constrained by moral disengagement processes (Graça et al., 2014; Piazza et al., 2015; Piazza and Gregson, 2025).

## Affective experiences at the heart of change

Surprisingly, only a few studies in this Research Topic addressed Empathy as one of the possible predictors of positive human interactions with nature, whether in the form of PEB (Nicolai et al.), intention to engage in it (Wang et al.), or understanding the importance of protecting species and ecosystems and reinforcing environmental education efforts (Costa et al., Lovati et al.). While Lovati et al. validated an Italian version of Tam's (2013) Dispositional Empathy with Nature scale, broadening the scope for exploring this variable and its relation to other forms of empathy, Nicolai et al.'s study examined how trait empathy and justice sensitivity contribute to the prediction of PEB. The findings revealed a direct link between justice sensitivity (but not empathy) and PEB; however, when cognitive and affective empathy were included together with justice sensitivity in a predictive model, affective empathy became the primary—and only significant—predictor, raising questions about how these factors interact in explaining PEB, and about how different conceptual approaches to empathy may also influence predictions. For example, disentangling empathic concern from affective empathy (which also entails empathic distress) may be useful to understand why affective empathy predicts PEB only when other “more cognitive” variables are in the equation.

The studies by Costa et al. and Wang et al. which examined zoo visitors and Natural Heritage tourism, respectively, explored the role of empathy in specific conservation targets. In both studies, questionnaires were administered to visitors of the sites of interest. In the zoo study, the affective experience of empathy was inferred rather than directly measured among visitors. A stronger emotional connection with animals was linked to greater concern for species conservation; however, the source of this connection was not related to visit frequency. The authors highlighted the potential of highly charismatic species—such as apes—to foster conservation concerns and support for zoo-led initiatives. A heated debate is currently ongoing about the effectiveness of captive settings in eliciting empathy and promoting engagement in habitat conservation. Natural heritage tourism popularity is growing and the researchers departed from the premise that it can foster empathy with nature and increase willingness to support conservation efforts. Focusing on a Scenic Area and forest in China, the authors confirmed this premise but also identified a modulating effect of awe on the relationship between empathy with nature and intentions to engage in PEBs, such as donations, support for sustainable products, and ethical environmental conduct.

Two articles focused on the effect of specific discrete emotions on nature connectedness and PEB. These address the role of empathy in bolstering ethical and eco-friendly behavior, an approach that stems from the vast evidence indicating that emotional experiences are key drivers of motivation (Panksepp, 1998; Khine, 2024) and decision-making (Bechara, 2004; Wang et al., 2024); emotions are often the primary source of moral decisions (Decety and Cowell, 2015), while conscious thought articulates the rationale to justify preconscious automatic decisions, for example by evoking socially accepted norms to justify those decisions (Haidt, 2001).

The experience of Awe was central to one theoretical article (Schilhab and Esbert), while Gratitude and Admiration were measured in two experimental studies (Che et al.) framed in Discrete Emotion Theory. The latter analyzed emotion interactions with self-transcendence and self-enhancement values in eco-friendly consumer choices. By activating different values in each induced emotion, the researchers were able to observe that when self-transcendence values are in focus, gratitude increases the likelihood of engaging in sustainable consumption. Conversely, when self-enhancement values are emphasized, it is admiration that increases consumers' motivation to consume sustainably. This relationship was mediated by self-efficacy. Schilhab and Esbert navigated multidisciplinary evidence to demonstrate how observations of wild animals and their purposefulness in their natural environment stimulate emotions and cognitions that are significant for the development of nature connectedness and potentially PEBs, creating a framework in support of immersive eco-education. When validating Tam's (2013) Dispositional Empathy with Nature scale, Lovati et al. found that this trait strongly predicts connectedness with nature and appears to have a protective effect against moral disengagement. These findings highlight the need to foster a connection with nature via emotionally engaging environmental education that cultivates empathy and responsibility toward nature to circumvent moral disengagement and achieve PEB.

## Values, norms, attitudes, intentions, and goals

A substantial body of articles delved into conscious cognitive processes and social influence in shaping pro-environmental or ethical behavior. This is an interesting outcome, consistent with Donald's (2022) view that efforts to promote PEBs still rely heavily on the assumption that conscious processes drive the change. The majority of models, rooted in social psychology, reflect this emphasis—an orientation also evident in the third cluster of articles presented here.

Using the widely applied Theory of Planned Behavior as their framework, three studies addressed college students' motivation to adopt low-carbon behaviors (Ren et al.), city residents' use of e-bikes (Wang and Xu), and the impact of social Media on Taiwanese Pro-environmental behavior (Liao). Across these studies, behavior is primarily driven by attitudes and intentions, shaped by perceived usefulness, social norms, and environmental awareness. Social norms showed inconsistent strength, as they were found to be less influential in the social media study. The latter study also showed that not all social media use is equally effective and that mere time spent online does not necessarily enhance environmental attitudes–rather, engagement with specific content is key. Given that information - in all these studies - played a key role in the development of attitudes, and those attitudes predict behavior via intention, closer attention is required to the content people have access to and engage with in the various media.

With Green travel behaviors (e.g., greater use of public transportation) as the target PEB of Zhang et al., Social Learning Theory framed the questions of how children can influence their parents' PEBs and specifically whether they can encourage them to choose greener travel options and how this process works. This study was designed to counteract previous studies that primarily approached behavior change intention by activating norms or changing beliefs (as in the Theory of Planned Behavior) because, despite people's intentions to travel green, they largely fail to do so. A field experiment using a pre–post design, which entailed delivering an educational intervention on green travel delivered to children, significantly increased parents' green travel behavior (as self-reported) while enhancing parents' environmental concerns and commitment, indicating that children are effective agents of change in their parents' PEBs.

Cruelty-free consumption was examined by Enginkaya and Saǧlam, specifically the role of moral values (e.g., concern for animals), social influences, and economic factors (e.g., price fairness). Using a mixed-methods design grounded in the Stimulus–Organism–Response (S–O–R) model and moral and social identity theories, the study showed that altruistic motives drive ethical preferences. Individuals concerned with animal welfare are more likely to choose cruelty-free products (displaying the leaping bunny logo), but ethical intentions are only enacted when prices are perceived as fair. Other factors emerged as important reinforcers: consumer choices generate psychological rewards, such as self-expression, empowerment, and social connection, and social support further steers this ethical behavior.

## Community spirit in eco-friendly action

Finally, two studies shifted the focus from individual goals and processes to the wider range of interpersonal and collective dimensions that bolster PEBs. A systematic review by Sarabi et al. showed that individuals are more likely to act sustainably when they perceive themselves as part of a group working toward a common objective (the “we-mode”), highlighting the need for group-based climate strategies. Another study (by Xin et al.), surveying urban consumers in China, examined how interpersonal harmony (a Confucian cultural value) influences people's eco-friendly buying and other sustainable lifestyle choices and found that interpersonal harmony promotes sustainable consumption via ethical thinking and personal norms, with environmental knowledge reinforcing these effects.

## Conclusion

From these contributions, we conclude that social cognitive theories for pursuing behavioral change are still dominant. However, the importance of emotions is evident, and we believe that we will see more of it in the PEB-fostering endeavors, especially at a time when we realize that many of the traditional approaches to behavior change are failing (Albarracín et al., 2024).

An unconscious affective process—distinct from emotions—that may also help explain why behavior change does not occur and thereby inaction prevails is a strong bind to habits (Verplanken and Orbell, 2019). However, none of the studies in this Research Topic addressed this factor.

A prevalent theme in all the studies in this Research Topic attempting to measure PEB is that researchers keep on using self-report measures, despite the known shortcomings of this approach (Lange, 2023). This approach relies on respondents' memory and suggested actions, does not control for social desirability, and does not embrace the challenge of measuring actual behavior.

We have identified key takeaways for educational settings, environmental campaigners, and policymakers: immersion, inducing emotion, well-curated information for engagement, value activation and attitude change, *involving* children through parental social learning.
